# Modified Two-Stage Exchange for Periprosthetic Joint Infection in UKA

**DOI:** 10.1155/2020/8860433

**Published:** 2020-08-14

**Authors:** Andrew G. Yun, Marilena Qutami, Kory B. Dylan Pasko

**Affiliations:** Center for Hip and Knee Replacement, Providence Saint John's Health Center, 2001 Santa Monica Blvd, Santa Monica, CA 90404, USA

## Abstract

Periprosthetic joint infection (PJI) is a rare complication following unicompartmental knee arthroplasty (UKA), and current management guidelines are still evolving. This report presents a novel surgical technique of resection arthroplasty with an articulated hemispacer as part of a 2-stage exchange protocol. A 66-year-old man developed a culture-negative PJI four months after a medial UKA. Rather than conventional full resection arthroplasty, the patient underwent partial resection with preservation of the lateral and patellofemoral compartments to maintain vascularized bone stock. An articulating hemispacer fashioned from the old implants after sterilization was reimplanted medially to preserve function during the course of antibiotic treatment. After successful eradication of infection, the patient underwent an uncomplicated conversion total knee replacement facilitated by prior preservation of bone stock. No stems or augments were needed. Therefore, a partial resection arthroplasty with an articulating hemispacer used in a 2-stage exchange protocol may be a reasonable option to eradicate infection and maintain function. In future cases of infected UKA, this technique warrants further consideration and investigation.

## 1. Introduction

Periprosthetic joint infection (PJI) is a devastating complication in the setting of knee arthroplasty. Infection in total knee arthroplasty (TKA) is one of the leading causes of failure, and the efficacy of treatment options for PJI in total knee arthroplasty is well-outlined [1]. Fortunately, the rate of infection in unicompartmental knee arthroplasty (UKA) is comparably lower, reportedly ranging from 0.1% to 0.8% [2].

Given the rarity of an infected UKA and limited experience with it, the options for treatment are still unclear. Attempts at management include surgical debridement, antibiotics, and implant retention (DAIR), one-stage exchange with conversion TKA, and two-stage exchange with conversion TKA [2–4]. Initial reports from the Mayo Clinic Registry suggest only limited success with DAIR, but higher rates of infection-free survival by two-stage exchange with conversion TKA [4]. The conventional surgical technique involves completing femoral and tibial cuts for subsequent TKA and exchange with a static spacer [4].

We present a modified two-stage exchange used in the management of an acute postoperative PJI after medial UKA. Rather than finishing the native knee with complete bone cuts for an eventual TKA, the existing anatomy of the patellofemoral joint and lateral compartment were preserved to maintain their bone stock and vascularity. An articulating hemispacer secured with antibiotic loaded cement in the medial compartment was used to maintain motion. In light of the successful treatment of this patient, the technique of aggressive debridement with preservation of bone stock, motion, and function may be a reasonable option for this infrequent yet challenging complication.

## 2. Case Presentation

**Figure 1:**
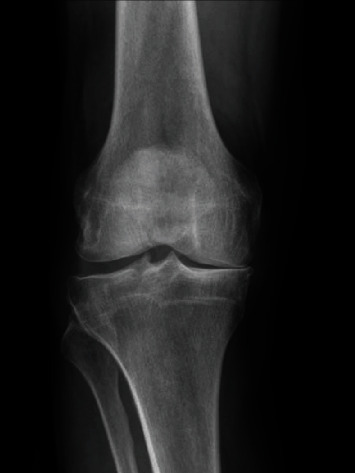
Preoperative radiograph with isolated medial disease.

**Figure 2:**
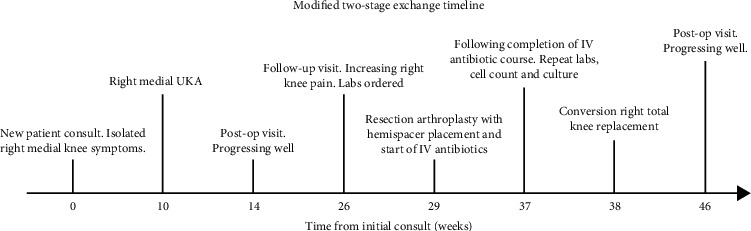
Acute timeline of modified two-state exchange for periprosthetic joint infection in UKA. Time not to scale.

A 66-year-old healthy man presented with two years of severe medial knee pain. Symptoms were refractory to conservative treatment including activity modification, physical therapy, injections, and over-the-counter medication. The patient had a history of arthroscopic medial meniscectomy eight years previously. On exam, he had an antalgic gait without a thrust, and his knee demonstrated correctable varus deformity, focal medial joint line tenderness, and stable cruciate ligaments. Weight-bearing radiographs revealed severe, isolated medial compartment arthritis (Figure 1). After a discussion of treatment options, the patient requested to proceed with UKA. A timeline of his care shows his course of treatment (Figure 2).

**Figure 3:**
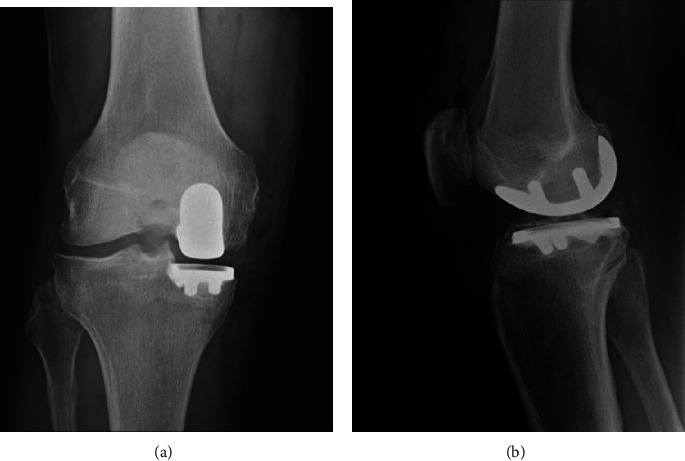
Unicompartmental knee arthroplasty in anatomic alignment. (a) Anteroposterior radiograph. (b) Lateral radiograph.

The patient underwent an uncomplicated right medial UKA (Figures 3(a) and 3(b)). He was discharged home the same day of surgery without event. His immediate postoperative course was unremarkable; his pain was well controlled, and his range of motion had returned to 125 degrees of flexion by 4 weeks.

Approximately 4 months after surgery, however, the patient returned with new complaints of swelling and increasing knee pain. On exam, he was afebrile, but his knee was tender with a significant effusion. Aspiration of his knee revealed cloudy fluid with an elevated white blood cell count (WBC) of 28,000 cells and a left shift with 90% polymorphonuclear (PMN) cells. Lab serologies showed an erythrocyte sedimentation rate (ESR) of 18 mm/hr and C-reactive protein (CRP) of 1.8 mg/L. Aerobic and anaerobic cultures showed no growth. With these laboratory findings, the patient was diagnosed with a culture-negative PJI by the modified MSIS criteria [5].

**Figure 4:**
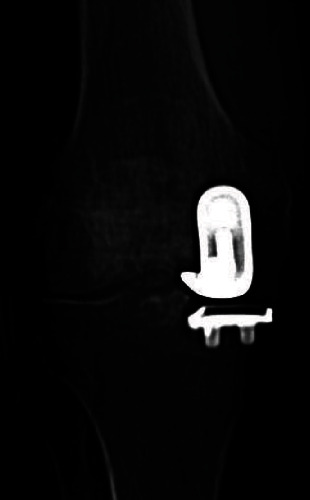
An articulating hemispacer fashioned from the old implants.

The patient was brought back to the operating room for a first stage implant resection and aggressive debridement with synovectomy. The implants were explanted without bone loss, and the medial posterior capsule was cleaned. Periprosthetic tissue samples were sent for histopathologic and microbiologic analysis. Fluid for culture from sonicated implants was not obtained. The femoral and tibial components were cleared of cement and sterilized in the autoclave as described by Hoffmann et al. [6]. Cement loaded with 2.4 grams of tobramycin and 4 grams of vancomycin was used to provisionally secure the implants, and a new polyethylene insert was placed (Figure 4). The patient was placed in a hinged knee brace locked from 0 to 90 degrees and allowed to proceed with weight bearing as tolerated.

Histopathologic study of the frozen sections intraoperatively showed greater than 50 neutrophils per high power field. The Gram stain showed 3+ neutrophils with no organisms. A second set of intraoperative cultures from retrieved tissue again showed no growth over 14 days, and the patient was treated as a culture-negative PJI. The infectious disease team began empirical treatment with intravenous vancomycin and ceftriaxone for 6 weeks.

**Figure 5:**
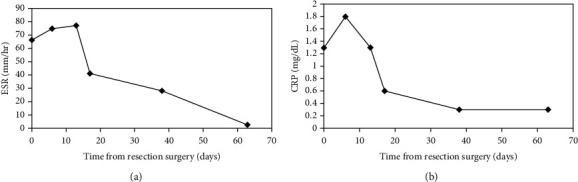
Lab serologies over time. (a) ESR vs. days after resection. (b) CRP vs. days after resection.

A detailed time analysis of the patient's serologies is revealing (Figures 5(a) and 5(b)). After the spacer was placed, the ESR and CRP continued to rise steeply and alarmingly for the first one to two weeks. Thereafter, the lab values began to decline steadily, and by eight weeks, both labs had reached normal values. Aspiration of synovial fluid two weeks after antibiotics were completed showed a new WBC count of 200 cells/microliter and 44% PMNs. All cultures remained negative.

**Figure 6:**
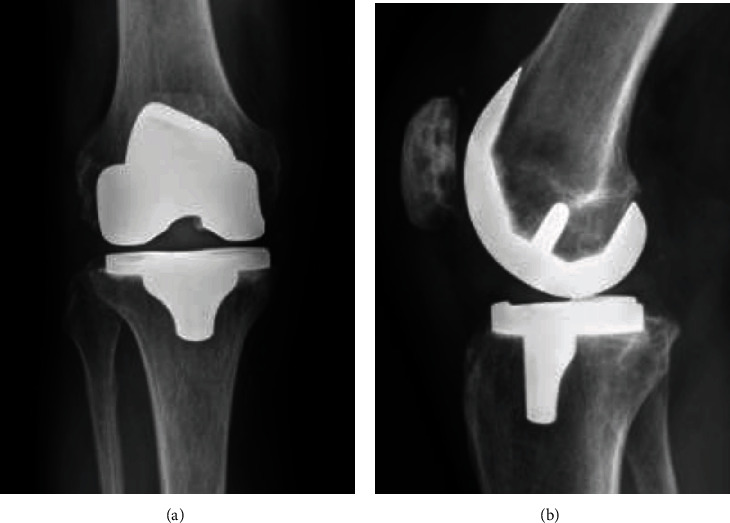
Conversion TKA after second stage exchange. (a) Anteroposterior view. (b) Lateral view.

The patient was then brought back at week nine for a second stage conversion TKA. It was noted that the soft tissues were flexible, exposure was routine, the spacer was removed without difficulty, and frozen sections were normal. As the patellofemoral and lateral compartment had been preserved, there was very little bone loss except for the prior medial compartment changes. Natural landmarks needed to establish femoral component rotation and joint line height were readily available. Unlike a typical reimplantation after prior resection arthroplasty, no additional augments or stems were needed. Primary instrumentation was used to implant a cruciate-substituting device with a 10 mm polyethylene insert (Figures 6(a) and 6(b)). All antibiotics were discontinued after 24 hours.

The patient recovered without further incident. He attained 0-125 degrees of motion in the four weeks following surgery. Four months after conversion TKA, he reported minimal to no pain and had resumed surfing. At 1-year follow-up, his range of motion is up to 140 degrees and his KOOS, JR score is 92. He consented to share his case history.

## 3. Discussion and Conclusion

Although comparatively rare, the presentation and management of PJI in UKA are becoming clearer. A majority of infections present acutely. Chalmers et al. reported the initial presentation at a mean of 6.4 months after primary UKA, with 67% of infections presenting within the first 4 weeks [2]. Similarly, Hernandez et al. found that 11 of 15 patients (73%) had become symptomatic within 90 days of the original surgery [4]. Our patient presented with new complaints of painful swelling four months after surgery, but we did not expect an infection *a priori* as we had not previously encountered PJI in our UKA population. We were startled by the abnormal serologies and the elevated synovial WBC count that confirmed the diagnosis of PJI by MSIS criteria [5].

The literature on managing PJI in UKA is sparse, and the established guidelines remain unclear. The overall rate of treatment failure is disappointing, as the rate of reinfection is reported to be 24% to 29% at 1 year [2, 4]. A deeper stratification suggests that DAIR carries the greatest risk of treatment failure between 38% by Chalmers et al. and 39% by Hernandez et al. [2, 4]. Further, those knees that are successfully cleared of infection with this technique may be at increased risk of opposite compartment degeneration. Several authors have reported subsequent revision TKA for progression of arthritic change in the opposite compartment [2, 4, 7].

Resection arthroplasty with conversion TKA at reimplantation, therefore, may be more successful than debridement and implant retention. In two limited series, the eradication of infection was reported in 4 of 4 patients by Hernandez et al. at 5 years, and in 3 of 4 patients by Chalmers et al. at 2 years [2, 4]. The dilemma then centers around the surgical management of the remaining portions of the native knee, the type of antibiotic spacer, and 1- or 2-stage exchange.

At the time of UKA explantation, prior studies report a surgical technique of complete removal of femoral and tibial bone to prepare the knee in a manner commonly done for PJI in TKA [2, 4, 7]. While technically familiar, this technique reduces bone stock that renders later reconstruction more challenging. It also removes vascularized bone and tissue that may assist in eradication of the infection. As can be inferred from treatment of septic arthritis in the native knee, success without bone removal has already been demonstrated with open irrigation and debridement (I and D) alone, arthroscopic I and D, and even serial arthrocentesis [8–10]. Theoretically, the natural surfaces of the knee may be comparatively more resistant to persistent biofilms, and the remaining viable and vascular tissues may support increased local antibiotic delivery. In the absence of established guidelines, our thought process in this patient was to minimize the amount of foreign body, to preserve vascularized tissues capable of eradicating infection, and to maximize bone stock for eventual conversion TKA.

Another objective of PJI management is to maintain function during the interval of treatment. Controversy between articulating and static spacers still exists with regard to success in curing infection; however, a review by Wyles and Abdel reported higher range of motion, less patella baja, and fewer complications with an articulating spacer [7]. While acknowledging that both static and articulating spacers work well, we chose to use the old components after sterilization in an attempt to preserve flexion and gait. Importantly, our patient was able to return to work during the two-month interval of antibiotic treatment.

A recognized alternative for PJI management in UKA is a 1-stage exchange. Labruyere et al. demonstrated success in 9 patients at 5 years [3]. In TKA, both 1- and 2-stages have been shown to be similarly effective [11]. We chose to proceed with a 2-stage protocol in this case because we were more familiar with the sequential method from our experience in managing infected TKA's and because we would have more certainty that the knee was aseptic before conversion TKA.

A close analysis of the trajectory of laboratory findings in this patient may also be enlightening. To our alarm, the ESR and CRP values became successively worse after debridement and spacer placement. As the ESR rose weekly from 66 mm/hr to 75 mm/hr and then to 77 mm/hr, we grew concerned over the possibility of early failure with the ongoing infection (Figures 5(a) and 5(b)). After discussion with our infectious disease consultant, it was decided to continue ongoing treatment. Fortunately, the increasing laboratory values plateaued and returned to normal by eight weeks. Follow-up aspiration of the knee showed no further evidence of infection. Understanding that such serologies may become worse before becoming better may be reassuring to surgeons.

Additionally, no specific organism could be cultured. While the diagnosis of PJI was inferred by clinical and laboratory parameters, microbiologic analysis in this patient with multiple fluid and periprosthetic tissue cultures was inconclusive. Adjunctive techniques to improve the identification of an infecting organism should be considered even though not used in this case report. To potentially reduce false negative culture results, a study by Sambri et al. reported that the culture of fluid from explanted components treated with either sonication or dithiothreitol improved sensitivity compared to routine tissue culture [12]. Alternatively, Zannoli et al. and Sambri et al. demonstrated that polymerase chain reaction (PCR) assays could also be used to assist in the timely identification of organisms [13, 14].

The rarity of PJI in UKA is comforting but also creates uncertainty over recognition, management, and course of disease. As the adoption of UKA continues to grow, a greater understanding of how to manage infections successfully becomes even more essential. In this case report, we offer the possibilities of infection eradication, preservation of interval patient function, and ease of eventual conversion TKA using a 2-stage protocol with an articulated hemispacer.

## Data Availability

Available per request.

## Abbreviations

PJI: Periprosthetic joint infection
TKA: Total knee arthroplasty
UKA: Unicompartmental knee arthroplasty
DAIR: Debridement, antibiotics, and implant retention
PMN: Polymorphonuclear
ESR: Erythrocyte sedimentation rate
CRP: C-Reactive protein
I and D: Irrigation and debridement
PCR: Polymerase chain reaction.
